# Versatile and declarative dynamic programming using pair algebras

**DOI:** 10.1186/1471-2105-6-224

**Published:** 2005-09-12

**Authors:** Peter Steffen, Robert Giegerich

**Affiliations:** 1 Faculty of Technology, Bielefeld University, Postfach 10 01 31, 33501 Bielefeld, Germany

## Abstract

**Background:**

Dynamic programming is a widely used programming technique in bioinformatics. In sharp contrast to the simplicity of textbook examples, implementing a dynamic programming algorithm for a novel and non-trivial application is a tedious and error prone task. The algebraic dynamic programming approach seeks to alleviate this situation by clearly separating the dynamic programming recurrences and scoring schemes.

**Results:**

Based on this programming style, we introduce a generic product operation of scoring schemes. This leads to a remarkable variety of applications, allowing us to achieve optimizations under multiple objective functions, alternative solutions and backtracing, holistic search space analysis, ambiguity checking, and more, without additional programming effort. We demonstrate the method on several applications for RNA secondary structure prediction.

**Conclusion:**

The product operation as introduced here adds a significant amount of flexibility to dynamic programming. It provides a versatile testbed for the development of new algorithmic ideas, which can immediately be put to practice.

## Background

Dynamic Programming is an elementary and widely used programming technique. Introductory textbooks on algorithms usually contain a section devoted to dynamic programming, where simple problems like matrix chain multiplication, polygon triangulation or string comparison are commonly used for the exposition. This programming technique is mainly taught by example. Once designed, all dynamic programming algorithms look similar: They are cast in terms of recurrences between table entries that store solutions to intermediate problems, from which the overall solution is constructed via a more or less sophisticated case analysis. However, the simplicity of these small programming examples is deceiving, as this style of programming provides no abstraction mechanisms, and hence it does not scale up well to more sophisticated problems.

In biological sequence analysis, dynamic programming algorithms are used on a great variety of problems, such as protein homology search, gene structure prediction, motif search, analysis of repetitive genomic elements, RNA secondary structure prediction, or interpretation of data from mass spectrometry [1-3]. The higher sophistication of these problems is reflected in a large number of recurrences – sometimes filling several pages – using more complicated case distinctions, numerous tables and elaborate scoring schemes. Hence, implementing a novel dynamic programming algorithm is a cumbersome task and requires extensive testing, while the resulting programs are difficult to re-use on related problems.

However, these difficulties are alleviated somewhat by a certain programming discipline: We may organize a dynamic programming algorithm such that the typical dynamic programming recurrences describe the problem decomposition and case analysis, but are completely separated from the intended optimization objective. Neither the initialization values for trivial problems, nor the scoring and objective functions, nor the required number of answers, not even the data type of the result must be visible in the recurrences. In this setting, one can simply exchange one encapsulated scoring scheme by another – including ones that do not solve optimizations problems, but compute other types of useful information about the search space. The new technique proposed here is a "product" operation on scoring schemes, creating a new scheme from two given ones. This product uses a non-trivial way to combine the two objective functions. Given some standard scoring schemes and the product operation, we can perform a remarkable variety of applications, such as optimizations under multiple objective functions, alternative solutions and backtracing, holistic search space analysis, ambiguity checking, and more, without additional programming effort, and without creating a need of debugging.

## Overview

We set the stage for our exposition with a condensed review of the "algebraic" approach to dynamic programming. We also introduce the individual scoring schemes that will be used in products later. We then introduce and discuss our definition of the product operation. From there, we proceed with a series of examples demonstrating the versatile use of products. The new product operation has been implemented and made available via the Bielefeld Bioinformatics Server [4], where the reader may run the examples presented in this paper, as well as his or her own ones. In our own, real-world programming projects, the product operation has become indispensable, and we report from our experience in the implementation of several recent bioinformatics tools.

## Algebraic dynamic programming by example

For our presentation, we need to give a short review of the concepts underlying the algebraic style of dynamic programming (ADP): trees, signatures, tree grammars, and evaluation algebras. We strive to avoid formalism as far as possible, and give an exemplified introduction here, sufficient for our present concerns. See [5] for a complete presentation of the ADP method. As a running example, we use the RNA secondary structure prediction problem. We start with a simple approach resulting in an ADP variant of Nussinov's algorithm [6] and move on to a more elaborate example to permit the demonstration of our new concepts. Nothing of the new ideas presented here is specific to the RNA folding problem. Products can be applied to all problems within the scope of algebraic dynamic programming, including pairwise problems like sequence alignment [5].

### RNA secondary structure prediction

**Figure 1:**
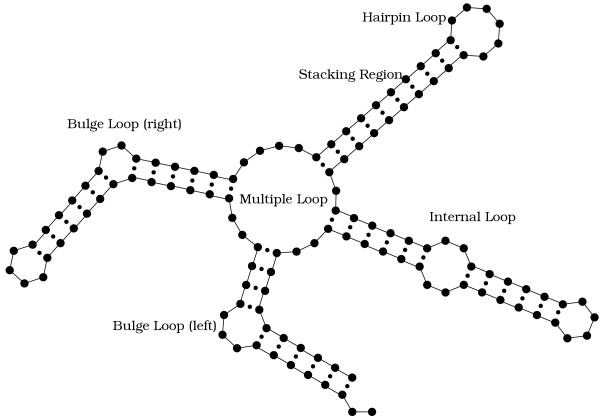
Typical elements found in RNA secondary structure.

While today the prediction of RNA 3D structure is inaccessible to computational methods, its *secondary structure*, given by the set of paired bases, can be predicted quite reliably. Figure 1 gives examples of typical elements found in RNA secondary structure, called stacking regions (or helices), bulge loops, internal loops, hairpin loops and multiple loops.

The first approach to structure prediction was proposed by Nussinov in 1978 and was based on the idea of maximizing the number of base pairs [6]. Today's algorithms are typically based on energy minimization.

### ADP methodology

When designing a dynamic programming algorithm in algebraic style, we need to specify four constituents:

• Alphabet: How is the input sequence given?

• Search space: What are the elements of the search space and how can they be represented?

• Scoring: Given an element of the search space, how do we score it?

• Objective: Given a number of scores, which are the ones we are interested in?

In the following, we will work through these steps for the RNA secondary structure prediction problem.

#### Alphabet

The input RNA sequence is a string over  = {*a*, *c*, *g*, *u*}.  is called the alphabet and * denotes the set of sequences over  of arbitrary length. *ε *denotes the empty string. In the following, we denote the input sequence with *w *∈ *.

#### Search space

**Figure 2:**
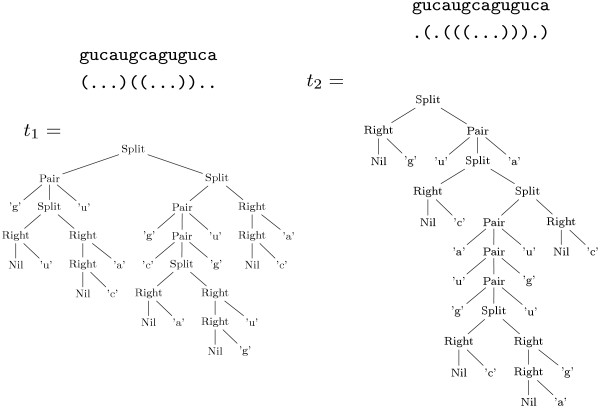
Two candidates in the search space for the best secondary structure for the sequence gucaugcaguguca.

Given the input sequence *w*, the search space is the set of all possible secondary structures the sequence *w *can form. In the ADP terminology, the elements of the search space for a given input sequence are called *candidates*. Our next task is to decide how to represent such candidates. Two possible ways are shown in Figure 2. The first variant is the well-known dot-bracket notation, where pairs of matching parentheses are used to denote pairing bases. The second variant, the tree representation, is the one we use in the algebraic approach.

Such a tree representation of candidates is quite commonly used in RNA structure analysis, but not so in other applications of dynamic programming. To appreciate the scope of the ADP method, it is important to see that such a representation exists for any application of dynamic programming (see appendix).

In our example, the trees are constructed using four different node labels. Each label represents a different situation, which we want to distinguish in the search space and in the eventual scoring of such a candidate. A node labeled *pair *represents the paring of two bases in the input sequence. The remaining nodes *right*, *split *and *nil *represent unpaired, branching and empty structures. It is easy to see that each tree is a suitable representation of the corresponding dot-bracket string. Also note that in each of the example trees, the original sequence can be retrieved by collecting the leaves in a counter-clockwise fashion. This is what we call the *yield *of the tree. The *yield function y *maps candidate trees back onto their corresponding sequences.

The next important concept is the notion of the *signature*. The signature describes the interface to the scoring functions needed in our algorithm. We can derive the signature for our current example by simply interpreting each of the candidate trees' node labels as a function declaration:



The symbol *Ans *is the abstract result domain. In the following, Σ denotes the signature, *T*_Σ _the set of trees over the signature Σ.

With the concepts of *yield *and *signature *we are now prepared to give a first definition of the search space: Given an input sequence *w *and a signature Σ, the search space *P*(*w*) is the subset of trees from *T*_Σ_, whose yield equals *w*. More formally, *P*(*w*) = {*t *∈ *T*_Σ_|*y*(*t*) = *w*}.

**Figure 3:**
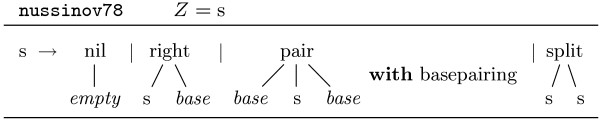
**Tree grammar nussinov78**. Terminal symbols in italics.

This would suffice as a very rough description of the search space. In general, we want to impose more restrictions on it, for example, we want to make sure, that the operator *pair *is only used in combination with valid base pairs. For this purpose we introduce the notion of *tree grammar*. Figure 3 shows grammar nussinov78, origin of our two example trees. This grammar consists of only one nonterminal, *s*, and one production with four alternatives, one for each of the four function symbols that label the nodes. *Z *denotes the axiom of the grammar. The symbols *base *and *empty *are terminal symbols, representing an arbitrary base and the empty sequence. The symbol *basepairing *is a syntactic predicate that guarantees that only valid base pairs can form a *pair*-node.

Our refined definition of the search space is the following: Given a tree grammar  over Σ and  and a sequence *w *∈ *, the language described by  is  = {*t*|*t *∈ *T*_Σ_, *t *can be derived from the axiom via the rules of }. The search space spawned by *w *is .

From the language theoretic viewpoint,  is the set of all parses of the sequence *w *for grammar .

The method we use for constructing the search space is called *yield parsing*, a solved problem that need not concern us here.

#### Scoring

Given an element of the search space as a tree *t *∈ , we need to score this element. In our example we are only interested in counting base pairs, so scoring is very simple: The score of a tree is the number of *pair*-nodes in *t*. For the two candidates of Figure 2 we obtain scores of 3 (*t*_1_) and 4 (*t*_2_). To implement this, we provide definitions for the functions that make up our signature Σ:



In mathematics, the interpretation of a signature by a concrete value set and functions operating thereon is called an algebra. Hence, scoring schemes are *algebras *in ADP. Our first example is the algebra bpmax for maximizing the number of base pairs. The subscript bpmax attached to the function names indicates, that these definitions are interpretations of the function under this algebra. In the following, we will omit these subscripts.

The flexibility of the algebraic approach lies in the fact that we don't have to stop with definition of *one *algebra. Simply define another algebra and get other results for the same search space. We will introduce a variety of algebras for our second, more elaborate example in Section *In-depth search space analysis*.

#### Objective

The tree grammar describes the search space, the algebra the scoring of solution candidates. Still missing is our optimization objective. For this purpose we add an objective function *h *to the algebra which chooses one or more elements from a list of candidate scores. An algebra together with an objective function forms an *evaluation algebra*. Thus algebra bpmax becomes:



A given candidate *t *can be evaluated in many different algebras; we use the notation *ε*(*t*) to indicate the value obtained from *t *under evaluation with algebra *ε*.

Given that yield parsing constructs the search space for a given input, all that is left to do is to evaluate the candidates in a given algebra, and make our choice via the objective function *h*. For example, candidates *t*_1 _and *t*_2 _of Figure 2 are evaluated by algebra bpmax in the following way:



**Definition 1 ***(Algebraic dynamic programming)*

• *An ADP problem is specified by a signature Σ over *, *a tree grammar **over Σ, and a Σ-evaluation algebra ε with objective function h*.

• *An ADP problem instance is posed by a string w *∈ *. *Its search space is the set of all its parses*, .

• *Solving an ADP problem is computing h*{ε(*t*) | *t *∈ } *in polynomial time and space with respect to |w|*.

In general, Bellman's Principle of Optimality [7] must be satisfied in order to achieve polynomial efficiency.

**Definition 2 **(*ADP formulation of Bellman's Principle*) *An evaluation algebra satisfies Bellman's Principle, if for each k-ary function f in *Σ *and all answer lists z*_1_,..., *z*_*k*_, *the objective function h satisfies*

*h*([*f*(*x*_1_,..., *x*_*k*_) | *x*_1 _← *z*_1_,..., *x*_*k *_← *z*_*k*_]) = *h*([*f*(*x*_1_,..., *x*_*k*_) | *x*_1 _← *h*(*z*_1_),..., *x*_*k *_← *h*(*z*_*k*_)])

as well as

*h*( *z *++ *z' *) = *h*( *h*(*z*) ++ *h*(*z'*) )

*where ++ denotes list concatenation, and ← denotes list membership*.

Bellman's Principle, when satisfied, allows the following implementation: As the trees that constitute the search space are constructed by the yield parser in a bottom up fashion, rather than building them explicitly as elements of *T*_Σ_, for each function symbol *f *the evaluation function *f*_*ε *_is called. Thus, the yield parser computes not trees, but their evaluations. To reduce their number (and thus to avoid exponential explosion) the objective function may be applied at an intermediate step where a list of alternative answers has been computed. Due to Bellman's Principle, the recursive intermediate applications of the objective function do not affect the final result.

As an example, consider the following two candidates (represented as terms) in the search space for sequence aucg:



Since algebra bpmax satisfies Bellman's Principle, we can apply the objective function *h *at intermediate steps inside the evaluation of candidates *t*_3 _and *t*_4_:



Given grammar and signature, the traditional dynamic programming recurrences can be derived mechanically to implement the yield parser. In the sequel, we shall use the name of a grammar as the name of the function that solves the dynamic programming problem at hand. Naturally, it takes two arguments, the evaluation algebra and the input sequence.

### In-depth search space analysis

Note that the case analysis in the Nussinov algorithm is redundant – even the sequence ' aa' is assigned the two trees Right (Right Nil 'a') 'a' and Split (Right Nil 'a') (Right Nil 'a'), which actually denote the same structure.

**Figure 4:**
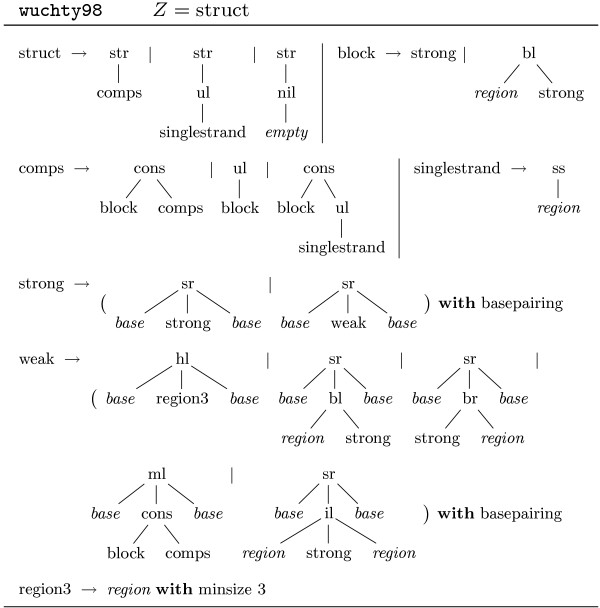
**Tree grammar wuchty98**. Terminal symbols in italics.

**Figure 5:**
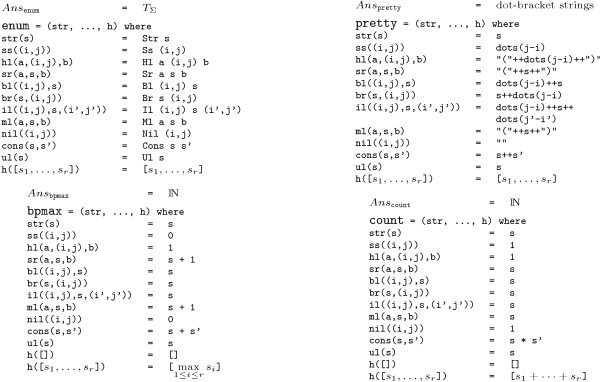
**Four evaluation algebras for grammar wuchty98**. Arguments a and b denote bases, (i, j) represents the input subword *w*_*i *+ 1 _.... *w*_*j*_, and s denotes answer values. Function dots(r) in algebra pretty yields a string of r dots ('.').

In order to study also suboptimal solutions, a non-redundant algorithm was presented in [8]. Figure 4 shows the grammar wuchty98. Here the signature has 8 function symbols, each one modeling a particular structure element, plus the list constructors (nil, ul, cons) to collect sequences of components in a unique way. Nonterminal symbol strong is used to capture structures without isolated (unstacked) base pairs, as "lonely pairs" are known to be energetically unstable. Purging them from the search space decreases the number of candidates considerably. This grammar, because of its non-redundancy, can also be used to study combinatorics, such as the expected number of feasible structures of a sequence of length *n*. This algorithm, as implemented in RNAsubopt [8], is widely used for structure prediction via energy minimization. The thermodynamic model is too elaborate to be presented here, and we will stick with base pair maximization as our optimization objective for the sake of this presentation. Figure 5 shows four evaluation algebras that we will use with grammar wuchty98. We illustrate their use via the following examples, where g(a,w) denotes the application of grammar g and algebra a to input w. Table 1 summarizes all results for an example sequence.

**Table 1:** Applications of grammars wuchty98 and nussinov78 with different individual algebras on input w = cgggauaccacu.

Application	Result
wuchty98(enum, w)	[Str (Ul (Bl (0,1) (Sr 'g' (Hl 'g' (3,10) 'c') 'u'))), Str (Ul (Bl (0,2) (Sr 'g' (Hl 'g' (4,10) 'c') 'u'))), Str (Cons (Bl (0,1) (Sr 'g' (Hl 'g' (3,7) 'c') 'c')) (Ul (Ss (9,12)))), Str (Cons (Bl (0,2) (Sr 'g' (Hl 'g' (4,7) 'c') 'c')) (Ul (Ss (9,12)))), Str (Ul (Ss (0,12)))]
wuchty98(pretty, w)	[".((.......))", "..((......))", ".((....))...", "..((...))...", "............"]
wuchty98(bpmax, w)	[2]
wuchty98(count,w)	[5]
nussinov78(count,w)	[9649270]

wuchty98(enum,w): The enumeration algebra enum yields unevaluated terms. By convention, function symbols are capitalized in the output. Since the objective function is identity, this call enumerates all candidates in the search space spawned by w. This is mainly useful in program debugging, as it allows us to inspect the search space actually traversed by our program.

wuchty98(pretty, w): The pretty-printing algebra pretty yields a dot-bracket string representation of the same structures as the above.

wuchty98(bpmax,w): The base pair maximization algebra is bpmax, such that this call yields the maximal number of base pairs that a structure for w can attain. Here the objective function is maximization, and it can be easily shown to satisfy Bellman's Principle. Similarly for grammar nussinov78.

wuchty98(count,w): The counting algebra count has as its objective function summation, and *ε*_*count*_(*t*) = 1 for all candidates *t*. Hence, summing over all candidate scores gives the number of candidates.

However, the evaluation functions are carefully written such that they satisfy Bellman's Principle. Thus, [length(wuchty98(enum,w))] == wuchty98(count,w), where the right-hand side is polynomial to compute, while the left-hand side typically is exponential due to the large number of answers returned by wuchty98(enum,w). Technically, the result of wuchty98(count,w) is a singleton list, hence the [...].

nussinov78(count,w): This computes (using an analogous version of the counting algebra not shown here) the number of structures considered by the Nussinov algorithm, which, in contrast to the above, is much larger than the size of the search space.

These examples show analyses achieved by individual algebras. We now turn to what can be done by their combination.

## Results and discussion

In this section we first introduce and discuss our definition of the product operation. From there, we proceed with a series of examples demonstrating its usage.

### The product operation on evaluation algebras

We define the product operation as follows:

**Definition 3 **(Product operation on evaluation algebras) Let M and N be evaluation algebras over Σ. Their product M***N is an evaluation algebra over Σ and has the functions

*f*_M***N_((*m*_1_, *n*_1_)...(*m*_*k*_, *n*_*k*_)) = (*f*_*M*_(*m*_1_,..., *m*_*k*_), *f*_*N*_(*n*_1_,..., *n*_*k*_)) for each *f *in Σ,

and the objective function



Above, ∈ denotes set membership and hence ignores duplicates. In contrast, ← denotes list membership and respects duplicates. Implementing set membership may require some extra filtering effort, but when the objective function *h*_*M*_, which computes *L*, does not produce duplicates anyway, it comes for free. We illustrate the application of the product operation to algebras bpmax and count:



Here, each function calculates a pair of two result values. The first is the result of algebra bpmax, the second is the result of algebra count. The interesting part is the objective function *h*. It receives the list of pairs as input, with each pair consisting of the candidate's scores for the first and for the second algebra. In the first step the objective function of algebra bpmax (max) is applied to the list of the first pair elements. The result is stored in *L*. In this example, *L *holds only one element, namely the maximum base pair score of the input list. In general, *L *holds many elements or may be empty. For each element of *L*, a new intermediate list is constructed that consists of all corresponding right pair elements of the input list. This intermediate list is then applied to the objective function of the second algebra (here: summation). Finally, the result of *h *is constructed by combination of all elements from *L *with their corresponding result for the second algebra stored in *r*. This computes the optimal number of base pairs, together with the number of candidate structures that achieve it.

One should not rely on intuition alone for understanding what M***N actually computes. For any tree grammar  and product algebra M***N, their combined meaning is well defined by Definition 1, and the view that a complete list of all candidates is determined first, with *h*_M***N _applied only once in the end, is very helpful for understanding. But does (M***N, *w*) actually do what it means? The implementation works correctly if and only if Bellman's Principle is satisfied by M***N, which is *not *implied when it holds for *M *and *N *individually! Hence, use of product algebras is subject to the following Proof obligation: Prove that M***N satisfies Bellman's Principle (Definition 2).

Alas, we have traded the need of writing and debugging code for a proof obligation. Fortunately, there is a theorem that covers many important cases:

**Theorem 1 ***(1) For any algebras M and N, and answer list x, (id*_M_* * * *id*_*N*_)(*x*) *is a permutation of x*.

*(2) If h*_*M *_*and h*_*N *_*minimize with respect to some order relations *≤_*M *_*and *≤_*n*_, *then h*_M***N _*minimizes with respect to the lexicographic ordering *(≤_*M*_, ≤_*N*_).

(3) If both M and N minimize as above and both satisfy Bellman's Principle, then so does M***N.

*Proof*. (1) According to Definition 3, the elements of *x *are merely re-grouped according to their first component. For this to hold, it is essential that *L *is treated as a set. (2) follows directly from Definition 3. (3) In the case of minimization, Bellman's Principle is equivalent to (strict) monotonicity of the functions *f*_*M *_and *f*_*N *_with respect to ≤_*M *_and ≤_*N*_, and this carries over to the combined functions (trivially) and the lexicographic ordering (because of (2)).     □

In the above proof, *strict *monotonicity is required only if we ask for multiple optimal, or for the *k *best solutions rather than a single, optimal one [9].

Theorem 1 (1) justifies our peculiar treatment of the list *L *as a set. It states that no elements of the search space are lost or get duplicated by the combination of two algebras. Theorem 1 (2,3) say that *** behaves as expected in the case of optimizing evaluation algebras. This is very useful, but not too surprising. A surprising variety of applications arises when *** is used with algebras that do not do optimization, like enum, count, and pretty.

The proof obligation is met for all the applications studied below. A case where the proof fails is, for example, wuchty98(count***count,w), which consequently delivers no meaningful result.

### Implementing the product operation

The algebraic style of dynamic programming can be practiced within any decent programming language. It is mainly a discipline of structuring our dynamic programming algorithms, the perfect separation of problem decomposition and scoring being the main achievement. When using the ASCII notation for tree grammars proposed in [5], the grammars can be compiled into executable code. Otherwise, one has to derive the explicit recurrences and implement the corresponding yield parser. Care must be taken to keep the implementation of the recurrences independent of the result data type, such that they can be run with different algebras, including arbitrary products.

All this given, the extra effort for using product algebras is small. It amounts to implementing the defining equations for the functions of M***N generically, i.e. for arbitrary evaluation algebras *M *and *N *over the common signature Σ. In a language which supports functions embedded in data structures, this is one line per evaluation function, witnessed by our implementation in Haskell (available for download). In other languages, abstract classes (Java) or templates (C++) can be used. It is essential to provide a generic product implementation. Otherwise, each new algebra combination must be hand-coded, which is not difficult to do, but tedious and error-prone, and hence necessitates debugging. A generic product, once tested, guarantees absence of errors for all combinations.

### Efficiency discussion

Before we turn to the uses of ***, a word on computational efficiency seems appropriate. Our approach requires to structure programs in a certain way. This induces a small (constant factor) overhead in space and time. For example, we must generically return a list of results, even with analyses that return only a single optimal value. Normally, each evaluation function is in *O*(1), and when *h *returns a single answer, asymptotic efficiency is solely determined by the tree grammar [5]. This asymptotic efficiency remains unaffected when we use a product algebra. Each table entry now holds a pair of answers, each of size *O*(1). Things change when we employ objective functions that produce multiple results, as the size of the desired output can become exponential in *n*, and then it dominates the overall computational effort. For example, the optimal base pair score may be associated with a large number of co-optimal candidates, especially when the grammar is ambiguous. Thus, if using *** makes our programs slower (asymptotically), it is not because of an intrinsic effect of the product operation, but because we decide to do more costly analyses by looking deeper into the search space.

The only exception to this rule is the situation where objective function *h*_*M *_produces duplicate values, which must be filtered out, as described with Definition 3. In this case, a non-constant factor proportional to the length of answer lists is incurred.

The concrete effect of using product algebras on CPU time and space is difficult to measure, as the product algebra runs a more sophisticated analysis than either single one. For an estimation, we measure the (otherwise meaningless) use of the same algebra twice. We compute wuchty98(bpmax,w) and compare to wuchty98(bpmax***bpmax,w). The outcome is shown in Table 2. For input lengths from 200 to 1600 bases, the product algebra uses 9.57% to 21.34% more space and is 18.97% to 29.46% slower than the single algebra.

**Table 2:** Measuring time and space requirements of the product operation. All results are for a C implementation of wuchty98, running on a 900 MHz Ultra Sparc 3 CPU under Sun Solaris 10. The space requirements were measured using a simple wrapper function for malloc, that counts the number of allocated bytes. Times were measured with gnu time.

	|w|	wuchty98(bpmax,w)	wuchty98(bpmax***bpmax,w)	%
time (sec)	200	0.58	0.69	+ 18.97
space (MB)	200	1.88	2.06	+ 9.57
time (sec)	400	4.65	6.02	+ 29.46
space (MB)	400	4.60	5.37	+ 16.74
time (sec)	800	52.04	65.54	+ 25.94
space (MB)	800	15.61	18.77	+ 20.24
time (sec)	1600	590.72	725.03	+ 22.74
space (MB)	1600	59.85	72.62	+ 21.34

### Applications of product algebras

We now turn to applications of product algebras. Table 3 summarizes all results of the analyses discussed in the sequel, for a fixed example RNA sequence.

**Table 3:** Example applications of product algebras with grammar wuchty98 on input w = cgggauaccacu.

Application	Result
wuchty98(bpmax***count,w)	[(2,4)]
wuchty98(bpmax***pretty,w)	[(2,".((.......))"), (2,"..((......))"), (2,".((....))..."), (2,"..((...))...")]
wuchty98(bpmax***enum,w)	[(2, Str (Ul (Bl (0,1) (Sr 'g' (Hl 'g' (3,10) 'c') 'u')))), (2, Str (Ul (Bl (0,2) (Sr 'g' (Hl 'g' (4,10) 'c') 'u')))), (2, Str (Cons (Bl (0,1) (Sr 'g' (Hl 'g' (3,7) 'c') 'c')) (Ul (Ss (9,12))))), (2, Str (Cons (Bl (0,2) (Sr 'g' (Hl 'g' (4,7) 'c') 'c')) (Ul (Ss (9,12)))))]
wuchty98(bpmax***(enum***pretty,w)	[(2,(Str (Ul (Bl (0,1) (Sr 'g' (Hl 'g' (3,10) 'c') 'u'))), ".((.......))")), (2, (Str (Ul (Bl (0,2) (Sr 'g' (Hl 'g' (4,10) 'c') 'u'))), "..((......))")), (2,(Str (Cons (Bl (0,1) (Sr 'g' (Hl 'g' (3,7) 'c') 'c')) (Ul (Ss (9,12)))), ".((....))...")), (2, (Str (Cons (Bl (0,2) (Sr 'g' (Hl 'g' (4,7) 'c') 'c')) (Ul (Ss (9,12)))), "..((...))..."))]
wuchty98(shape***count, w)	[("_ [_]", 2), ("_ [_]_", 2), ("_",1)]
wuchty98(bpmax(5)***shape, w)	[(2,"_ [_]"), (2,"_ [_]_"), (0,"_")]
wuchty98(bpmax(5)***(shape***count), w)	[(2, ("_[_]", 2)), (2, ("_[_]_", 2)), (0, ("_",1))]
wuchty98(shape***bpmax, w)	[("_[_]", 2), ("_[_]_", 2), ("_",0)]
wuchty98(bpmax***pretty', w)	[(2,".((....))...")]
wuchty98(pretty***count, w)	[(".((.......))",1), ("..((......))",1), (".((....))...",1), ("..((....))...",1), ("............",1)]

#### Application 1: Backtracing and co-optimal solutions

Often, we want not only the optimal answer value, but also a candidate which achieves the optimum. We may ask if there are several optimal candidates. If so, we may want to see them all, maybe even including some near-optimal candidates. The traditional technique is to store a table of intermediate answers and backtrace through the optimizing decisions made [1]. This backtracing can become quite intricate to program if we ask for more than one candidate. We can answer these questions without additional programming efforts using products:

wuchty98(bpmax***count,w) computes the optimal number of base pairs, together with the number of candidate structures that achieve it.

wuchty98(bpmax***enum,w) computes the optimal number of base pairs, together with all structures for w that achieve this maximum, in their representation as terms from *T*_Σ_.

wuchty98(bpmax***pretty,w) does the same as the previous call, producing the string representation of structures.

wuchty98(bpmax***(enum***pretty),w) does both of the above.

To verify all these statements, apply Definition 3, or visit the ADP web site and run your own examples. It is a nontrivial consequence of Definition 3 that the above product algebras in fact give *all *co-optimal solutions. Should only a single one be desired, we would use enum or pretty with a modified objective function *h *that retains only one element.

Note that our substitution of backtracing by a "forward" computation does not affect asymptotic runtime efficiency. With bpmax***enum, for example, the algebra stores in each table entry the optimal base pair count, together with the top node of the optimal candidate(s) and pointers to its immediate substructures, which reside in other table entries. Hence, even if there should be an exponential number of co-optimal answers, they are represented in polynomial space, because subtrees are shared. Should the user decide to have them all printed, exponential effort is incurred, just as with a backtracing implementation.

### Application 2: Holistic search space analysis

Abstract shapes were recently proposed in [10] as a means to obtain a holistic view of an RNA molecule's folding space, avoiding the explicit enumeration of a large number of structures. Bypassing all relevant mathematics, let us just say that an RNA shape is an object that captures structural features, like the nesting pattern of stacking regions, but not their size. We visualize shapes by strings alike dot-bracket notation, such as _[_[_]], where _ denotes an unpaired region and [together with the matching ] denotes a complete helix of arbitrary length. This is achieved by the following shape algebra. Here, function shMerge appends two strings and merges adjacent characters for unpaired regions (_). The function nub eliminates duplicates from its input list.



Together with a creative use of ***, the shape algebra allows us to analyze the number of possible shapes, the size of their membership, and the (near-) optimality of members, and so on. Let bpmax(k) be bpmax with an objective function that retains the best k answers (without duplicates).

wuchty98(shape***count,w) computes all the shapes in the search space spawned by w, and the number of structures that map onto each shape.

wuchty98(bpmax(k)***shape,w) computes the best k base pair scores, together with their candidates' shapes.

wuchty98(bpmax(k)***(shape***count),w)

computes base pairs and shapes as above, plus the number of structures that achieve this number of base pairs in the given shape.

wuchty98(shape***bpmax,w) computes for each shape the maximum number of base pairs among all structures of this shape.

### Application 3: Optimization under lexicographic orderings

Theorem 1 is useful in practice as one can test different objectives independently and then combine them in one operation. A simple case of using two orderings would be the following: Assume we have a case with a large number of co-optimal solutions. Let pretty' be pretty with *h *= *min*.

wuchty98(bpmax***pretty',w) computes among several optimal structures the one which comes alphabetically first according to its string representation.

Of course, there are more meaningful uses of a primary and a secondary optimization objective. For lack of space, we refrain from introducing another optimizing algebra here.

### Application 4: Testing ambiguity

Dynamic programming algorithms can often be written in a simpler way if we do not care whether the same solution is considered many times during the optimization. This does not affect the overall optimum. A dynamic programming algorithm is then called redundant or ambiguous. In such a case, the computation of a list of near-optimal solutions is cumbersome, as it contains duplicates whose number often has an exponential growth pattern. Also, search space statistics become problematic – for example, the counting algebra speaks about the algorithm rather than the problem space, as it counts evaluated, but not necessarily distinct solutions. Tree grammars with a suitable probabilistic evaluation algebra implement stochastic context free grammars (SCFGs) [2]. The frequently used statistical scoring schemes, when trying to find the answer of maximal probability (the Viterbi algorithm, cf. [2]), are fooled by the presence of redundant solutions. In principle, it is clear how to control ambiguity [11]. One needs to show unambiguity of the *tree *grammar in the language theoretic sense (not the associated *string *grammar – it is always ambiguous, else we would not have an optimization problem), and the existence of an injective mapping from *T*_Σ _to a canonical model of the search space. However, the proofs involved are not trivial. Rather, one would like to implement a check for ambiguity that is applicable for any given tree grammar, but this may be difficult or even impossible, as the problem is closely related to ambiguity of context free languages, which is known to be formally undecidable.

Recently, Dowell and Eddy showed that ambiguity really matters in practice for SCFG design, and they suggest a procedure for ambiguity testing [12]. This test uses a combination of Viterbi and Inside algorithms to check whether the (putatively optimal) candidate returned by Viterbi has an alternative derivation. A more complete test is the following, and due to the use of ***, it requires no implementation effort:

The required homomorphism from the search space to the canonical model may be coded as another evaluation algebra. In fact, if we choose the dot-bracket string representation as the canonical model, algebra pretty does exactly this. We can test for ambiguity by testing injectivity of pretty – by calling wuchty98(pretty***count,w) on a number of inputs w. If any count larger than 1 shows up in the results, we have proved ambiguity. This test is strictly stronger than the one by Dowell and Eddy, which detects ambiguity only if it occurs with the (sampled) "near-optimal" predictions. This and other test methods are studied in detail in [13].

#### Limitations of the product operation

The above applications demonstrate the considerable versatility of the algebra product. In particular, since a product algebra is an algebra itself, we can work with algebra triples, quadruples, and so on. All of these will be combined in the same fashion, and here we reach the limits of the product operation. The given definition of *** is not the only way needed to combine two algebras. In abstract shape analysis [10], we use three algebras mfe (computing minimal free energy), shape and pretty. A shape representative structure is the structure of minimal free energy within the shape. Similarly to the above, wuchty98(shape***(mfe***pretty),w) computes the representatives of *all *shapes. However, computing *only *the *k *best shape representatives requires minimization within and across shapes, which neither mfe***shape nor shape***mfe can achieve. Hence, a hand-coded combination of the three algebras is necessary for this particular analysis.

## Conclusion

We hope to have demonstrated that the evaluation algebra product as introduced here adds a significant amount of flexibility to dynamic programming. We have shown how ten meaningful analyses with quite diverse objectives can be obtained by using different products of a few simple algebras. The techniques we have shown here pertain not just to RNA folding problems, but to all dynamic programming algorithms that can be formulated in the algebraic style.

The benefits from using a particular coding discipline do not come for free. There is some learning effort required to adapt to a systematic approach and to abandon traditional coding habits. After that, the discipline pays back by making programmers more productive. Yet, the pay-off is hard to quantify. We therefore conclude with a subjective summary of our experience as bioinformatics toolsmiths. After training a generation of students on the concepts presented here, we have enjoyed a boost in programming productivity. Four bioinformatics tools have been developed using this approach -pknotsRG [14], RNAshapes [10], RNAhybrid [15] and RNAcast [16]. The "largest" example is the pseudoknot folding grammar, which uses 47 nonterminal symbols and a 140-fold case distinction. The techniques described here have helped to master such complexity in several ways:

• The abstract formulation of dynamic programming recurrences in the form of grammars makes it easy to communicate and refine algorithmic ideas.

• Writing non-ambiguous grammars for optimization problems allows us to use the same algorithm for mathematical analysis of the search space.

• Ambiguity checking ensures us that such analyses are correct, that probabilistic analyses are not fooled by redundant recurrences, and that near-optimal solutions when reported do not contain duplicates.

• enum algebras allow for algorithm introspection – we can obtain a protocol of all solution candidates the program has seen, a quite effective program validation aid.

• Using pretty and enum algebras in products frees us from the tedious programming of backtracing routines.

• The use of the product operation allows us the create new analyses essentially with three key strokes – and a proof obligation that must be met.

This has created a good testbed for the development of new algorithmic ideas, which can immediately be put to practice.

## Methods

The new product operation has been implemented and made available via the Bielefeld Bioinformatics Server [4], where the reader may run the examples presented in this paper, as well as his or her own ones.

## Appendix: "Reverse engineering" of dynamic programming algorithms

To support the claim that a tree representation of candidates always exists, we show how such a representation can be found by a sort of reverse engineering of a given DP algorithm not formulated in the ADP framework. The reasoning applied is called symbolic evaluation in computer science terminology.

Assume that the algorithm returns a score of (say) *X*_1_. The last step in the computation of this score value must have been the application of some function *f*_1_. This function corresponds to a particular problem decomposition. Let *X*_1 _= *f*_1_(*a*, *b*,..., *X*_2_, *X*_3_,...). Here *a*, *b*,... come from the input problem and are parameters for the local score contribution, while *X*_2_, *X*_3_,... are scores from subproblems. For example, in pairwise sequence alignment, *f*_1 _may correspond to replacing one amino acid by another, adding the PAM score of mutating *a *to *b *to the score of the remaining alignment, *X*_2_. There are no additional *X*_*i *_in this case. With RNA folding, *f*_1 _may correspond to a multiloop, with *a*, *b *being the closing base pair, and *X*_2 _corresponding to the first stem within the multiloop, and *X*_3 _to a succession of the other stems. This includes DP algorithms that consider RNA pseudoknots. *f*_1 _may correspond to composing a pseudoknot from two crossing helices corresponding to *X*_2 _and *X*_3_, as is apparent in the pseudoknot folding program *pknotsRG *[14], which was developed with the ADP method. Unless the DP algorithm has been written very systematically, the actual operations that implement *f*_1 _may be scattered over various places in the code.

Whatever the meaning and implementation of *f*_1 _is – we record the formula *F*_1 _= *f*_1_(*a*, *b*,..., *X*_2_, *X*_3_,...), and recursively apply the same consideration to the subproblem scores *X*_*i*_, using the appropriate functions *f*_*i *_to obtain formulas *F*_*i *_which we substitute in *F*_1 _for the corresponding *X*_*i*_, and so on. This eventually results in a large formula *F*_1 _that contains no more *X*_*i *_and hence depends only on the input parameters. We have recorded, as the symbolic expression *F*_1_, the complete computation of the score *X*_1_. We have not recorded how this formula was found – it does not at all reflect the control structure of the algorithm. As any formula can be naturally considered as a tree, we have found a tree representation of the optimal candidate. Clearly, all suboptimal candidates have similar representations, obtained in the same way.

The reverse engineering of a known DP algorithm is not only a theoretical possibility, but also an instructive educational exercise, and helpful in analyzing and improving upon previous work.

## Authors' contributions

Both authors cooperated closely in developing the work presented here, and in writing the manuscript.
