# Correction: versatile and declarative dynamic programming using pair algebras

**DOI:** 10.1186/1471-2105-7-214

**Published:** 2006-04-20

**Authors:** Peter Steffen, Robert Giegerich

**Affiliations:** 1 Faculty of Technology, Bielefeld University, Postfach 10 01 31, 33501 Bielefeld, Germany

We have located a typesetting error in our recent publication in BMC Bioinformatics 2005,6:224 [1].

Although the error is quite minor (*l*' = *l *has been miswritten as *l*' ∈ *L*), it occurs in the central Definition 3 in the paper, and is also pasted into subsequent example code (both error sites on p.7, right column).

This error unfortunately looks mathematically plausible, although it is in contradiction with the given verbal explanations.

The corrected Definition 3 now reads as:

**Definition 3 **(Product operation on evaluation algebras) Let *M *and *N *be evaluation algebras over Σ. Their product M***N is an evaluation algebra over Σ and has the functions

*f*_M***N _((*m*_1_, *n*_1_)...(*m_k_*, *n_k_*)) = (*f_M _*(*m*_1_,...,*m_k_*), *f_N_*(*n*_1_,...,*n_k_*)) for each *f *in Σ,

and the objective function

   *h*_M***N_([(*m*_1_, *n*_1_)...(*m_k_*, *n_k_*)]) = [(*l*, *r*)|

      *l *∈ *L*,

      *r *← *h_N_*([*r*'|(*l*', *r*') ← [(*m*_1_, *n*_1_)...(*m_k_*, *n_k_*)], *l*' = *l*])],

      where *L *= *h_M _*([*m*_1_,...,*n_k_*]).
